# Forced adaptation: plant proteins to fight climate change

**DOI:** 10.3389/fpls.2014.00762

**Published:** 2015-01-05

**Authors:** Ana E. Valdés

**Affiliations:** Physiological Botany, Uppsala BioCenter, Linnean Center for Plant Biology, Uppsala UniversityUppsala, Sweden

**Keywords:** Drought stress, HD-Zip transcription factors, ABA signaling pathway, climate adaptation, *Arabidopsis thaliana*

Increasing distress about climate change consequences is noticeable in daily press releases and science news articles. In the last 5 years more than 3 hundred thousand scientific articles included the terms “climate change,” “drought stress” and/or “climate adaptation” as a main topic. This build-up of energy devoted to understand climate change significance parallels the fact that any living organism must be able to cope with environmental changes to survive. Plant's sessile condition reinforces even more the need of an efficient adaptive response to counteract a suboptimal environment. Such adaptive strategies synchronize growth and development adjustments, as well as cellular and molecular activities, aimed at an efficient use of scarce resources, e.g., water.

Plant hormones are often involved as systemic mediators of the perception and integration of environmental cues. For instance, abscisic acid (ABA) accumulation upon drought perception serves as an initial signal for long-term acclimation reactions, which eventually involve the differential expression of genes leading to changes in transcript and protein patterns (Valdés et al., 2013). Plant-specific homeodomain leucine-zipper (HD-Zip) class I genes have been for long time suggested as players in the signal transduction to adjust growth and development under stress circumstances (Söderman et al., 1996; Olsson et al., 2004), but it was not until recently that their specific regulatory mechanism within the drought-induced ABA signaling pathway was discovered (Valdés et al., 2012). The authors reported two *Arabidopsis* HD-Zip I transcription factors, named ATHB7 and ATHB12, down-regulating a number of genes encoding ABA-receptor proteins, in addition to up-regulating protein phosphatases type 2C. Both ABA receptors and protein phosphatases 2C are well established components of the ABA signaling pathway (Santiago et al., 2009). This fine modulation of the stress perception confers the plant with the capacity to adapt to exposure to constant levels of ABA, thus causing the ABA response to be transient in character and providing the plant with the possibility to turn on and off the adaptive response at will. Stress adaptation is essential to evolutionary fitness and, as such, it has been discovered that a similar biological function is retained by orthologous HD-Zip I proteins in many plant species (Song et al., 2012; Zhao et al., 2014).

As previously mentioned, developmental changes and morphological alterations are part of the plant adaptation and, besides controlling stress responses, HD-Zip I genes have additional roles in controlling development (Ariel et al., 2010). An interesting point raised by Valdés et al. (2012) is that other HD-Zip genes sharing targets, but differing in expression patterns or dependence on specific external conditions may have similar functions in modulating the ABA signal perception. HD-Zip superfamily of transcription factors includes also class III, which are major polarity and patterning determinants (Prigge et al., 2005). A potential HD-Zip I/HD-Zip III antagonism in the control of ABA-receptor genes has been recently proposed (Brandt et al., 2014), manifesting that complex relationships between classes appear to lead the integration of environmental and developmental cues. Though class I and class III members do not seem to regulate the same ABA receptors (Liu et al., 2012; Valdés et al., 2012) it may be possible that both families oppositely regulate the expression of related genes in a cell-type specific manner (Brandt et al., 2014). Similar genetic interactions between proteins belonging to different class families have been described in the integration of shade escape control and leaf patterning (Brandt et al., 2012).

Besides water availability, the plant environmental context is defined by additional, simultaneous external factors, e.g., light and temperature, and should these factors influence the transcript levels of HD-Zip proteins the dynamic behavior of the ABA-driven stress response becomes automatically dependent on such factors. This implies that cross-communication between different signaling systems should be mediated by the same HD-Zip proteins. In this sense, available genomic and proteomic data have predicted *in silico* interactions between genes early regulated in the shade-avoidance response and, ATHB7 and ATHB12 that highlight their potential participation also in light signaling pathways (Ciolfi et al., 2013).

The intricate network established within this superfamily of transcription factors suggest that the plant-specific and evolutionary highly conserved HD-Zip proteins are crucial players modulating stress responses and may be linking patterning and adaptation by acting to adjust developmental programs to specific environmental situations.

## Conflict of interest statement

The author declares that the research was conducted in the absence of any commercial or financial relationships that could be construed as a potential conflict of interest.
